# Does Calomel Exert Any Specific Influence on the Biliary Secretion

**Published:** 1849-07

**Authors:** 


*Does Calomel exert any Specific Influence on the Biliary Secretion.*—By Michea.—M. Michea, after detailing the various opinions which have been advanced as to the nature of the green stools which so often ensue on the administration of calomel, states the results of his own examination of fecal matters under varying circumstances. He observes that in the dejections of healthy persons, the greater proportion of the elements of the bile exist only in a state of combination, and require alcohol and potassa for their detection. Free bile, i. e. bile soluble in water, is, according to Berzelius, found only in the proportion of 7-8 per cent. of fecal matters. For the detection of free bile M. Michea prefers nitric acid to Pettenkofer's test. In six persons, in good health, the acid furnished no traces of bile: and of three others suffering from gastro-intestinal affections. in one only wh[illegible] had bilious vomiting and green purging, was a notable proportion indicated. Of eight cases in which calomel was administered, in some large, in others in small doses for successive days, in four only were green stools produced. This shows that the action of the calomel on the bowels is very uncertain, and tends to confirm the truth of Mialhe's doctrines, that it is dependent upon the conversion of the chloride into deuto-chloride, most of the persons in whom it produced no action, having been women, whose humours contains less of the chlorides than do those of men. Not only are the stools changed in color, but they are so in consistency, possessing neither the solidity of the natural stool, nor the aqueousness of those of dysentery or typhoid fever, but assuming an intermediate viscous character. In two of these cases the acid plainly evinced the existence of bile; and not only of bilverdine, but of the albumen of that fluid. In the other two cases albumen was also precipitated, giving however a color more analogous to the bilivardine of Mulder. In stools produced by various other purgatives, in five individuals no traces of the bile were produced, nor did they assume the consistency of those induced by calomel. The general result of the investigation is, then, to confirm the opinion of those who maintain the agency of calomel on the biliary secretion—*L' Union Medicale.*

*Increase of the Cholera in Paris.*—We regret to state that the latest accounts from Paris, dated the 26th inst., represent the cholera as making alarming progress, especially among the patients in the public hospitals. In London the hospitals have been almost entirely free from the disease.

At the Saltpetriére, in Paris, there were in one day among the inmates, who are chiefly aged persons, no fewer than *fifty* cases.

There have been altogether in Paris since the 9th inst., 385 cases, and 180 deaths—a mortality of nearly fifty per cent. Of the 385 cases, 203 were among the hospital patients, and the remainder were received from without. There are at present but few convalescents.—*Ibid.*

---

Prize Essay.—The New York State Medical Society, at their recent meeting, passed the following resolutions:

*Resolved,* That a prize of twenty dollars be offered by this society for a tract of not less than four, or more than sixteen pages, which shall most clearly expose the pernicious influence of nostrums or secret remedies, upon the health and morals of the community.

*Resolved,* that the Committee on Prize Essays award the above prize, and publish the accepted communications.

The Chairman of the Prize Committee is Dr. James McNaughton of Albany.

